# Prospective validation of quantitative CEA mRNA detection in peritoneal washes in gastric carcinoma patients

**DOI:** 10.1038/sj.bjc.6602802

**Published:** 2005-10-04

**Authors:** S Ito, H Nakanishi, Y Kodera, Y Mochizuki, M Tatematsu, Y Yamamura

**Affiliations:** 1 Department of Gastroenterological Surgery, Aichi Cancer Center Hospital, Nagoya, Japan; 2 Division of Oncological Pathology, Aichi Cancer Center Research Institute, Nagoya, Japan; 3 Pathology and Molecular Diagnostics, Aichi Cancer Center Hospital, Nagoya, Japan; 4 Department of Surgery II, Nagoya University School of Medicine, Nagoya, Japan

**Keywords:** peritoneal metastasis, gastric cancer, real-time quantitative RT–PCR, prospective study

## Abstract

Prediction of peritoneal relapse is extremely important for gastric cancer patients after curative surgery. The present study prospectively validates the prognostic ability of quantifying carcinoembryonic antigen (CEA) mRNA in peritoneal washes by real-time reverse transcriptase–polymerase chain reaction. Based on a retrospective study of 197 curatively resected gastric cancer patients (training set), we determined a cutoff value of CEA mRNA using receiver-operating characteristic curve. We used this cutoff value to validate the risk of peritoneal recurrence in a new cohort of 86 gastric cancer patients (validation set) between July 2000 and December 2002 in a prospective study. During the median 30 months of postoperative surveillance, 20 of the 86 patients died, and 13 of the 20 developed peritoneal metastases. Peritoneal recurrence-free survival as well as overall survival was significantly worse in patients with positive CEA mRNA (*P*<0.0001). Multivariate analysis with the Cox proportional hazards model showed that positive CEA mRNA was a significant independent risk factor with both survival (*P*=0.0130) and peritoneal recurrence-free survival (*P*=0.0006) as end points. These results indicate that quantitation of CEA mRNA in peritoneal washes is a reliable prognostic indicator of peritoneal recurrence in the clinical setting.

Although the survival of patients with gastric cancer has improved due to the development of new diagnostic tools for early detection and the nationwide practice of mass screening, gastric carcinoma remains a leading cause of cancer death in Japan as well as in other countries. Peritoneal dissemination, the most frequent type of recurrence in patients with gastric cancer, is considered to arise from free cancer cells in the peritoneal cavity exfoliated from the serosal surface of the stomach penetrated by the primary tumour. Peritoneal washes have been cytologically examined at laparotomy as an international gold standard to detect such tumour cells and to evaluate the risk of peritoneal recurrence (Boku *et al*, 1990; Bonenkamp *et al*, 1996; Bando *et al*, 1999; Hayes *et al*, 1999; Kodera *et al*, 1999). The results of this procedure are recognised as an important prognostic determinant (Bonenkamp *et al*, 1996; Hayes *et al*, 1999); however, conventional Papanicolaou staining lacks sensitivity and some patients whose washes were cytologically negative develop recurrent disease in the peritoneal cavity after surgery (Abe *et al*, 1995). Although several investigators have reported that immunohistochemistry with antibody panels can be an aid to conventional cytology (Juhl *et al*, 1994; Benevolo *et al*, 1998), sensitivity still needs to be increased. We therefore applied the reverse transcriptase–polymerase chain reaction (RT–PCR) to detect micrometastases in the peritoneal cavity, using carcinoembryonic antigen (CEA) as the target gene (Nakanishi *et al*, 1997; Kodera *et al*, 1998). We then applied real-time RT–PCR to quantify free cancer cells in peritoneal washes (Nakanishi *et al*, 2000) and declared the prognostic significance of intra-abdominal CEA mRNA levels (Kodera *et al*, 2002). Since this report, many investigators have concurred that CEA mRNA levels represent an accurate retrospective determination of the risk of peritoneal recurrence in patients with gastric cancer (Yonemura *et al*, 2001; Fujii *et al*, 2002; Marutsuka *et al*, 2003; Tokuda *et al*, 2003; Ueno *et al*, 2003; Oyama *et al*, 2004). In breast cancer, the front lines of micrometastasis research, several prospective studies have demonstrated the prognostic significance of the detection of micrometastasis in the sentinel lymph node or circulating tumour cells in the blood in node-negative cancer patients (Gillanders *et al*, 2004; Jotsuka *et al*, 2004). However, such prospective quantitative studies of micrometastasis in patients with gastric cancer have not been reported.

Based on a cutoff value predetermined from retrospective studies between 1995 and 2000, we validated the ability of quantitative CEA mRNA detection in peritoneal washes to predict peritoneal recurrence in a prospective study between 2000 and 2004. We confirmed that levels of CEA mRNA can predict peritoneal recurrence in patients with gastric cancer who underwent curative resection and discuss its therapeutic application for selecting patients who might benefit from chemotherapy in the clinical setting.

## MATERIALS AND METHODS

### Study design

The study consisted of sequential training and validation subsets. The training set was a retrospective study of 197 patients with gastric cancer who underwent curative surgery at Aichi Cancer Center Hospital between April 1995 and March 1999. During this period, CEA mRNA expression was examined in peritoneal washes from 256 patients. Among these, 49 patients with liver and/or peritoneal metastases at laparotomy, nine with macroscopically residual disease and one with another active malignancy were excluded from this study. The cutoff value of CEA mRNA was determined from this study by analysing receiver-operating characteristic (ROC) curve based on CEA mRNA values and clinical follow-up data until April 2000 with a median follow-up period of 38 months. The validation set was a prospective cohort study to validate the ability of quantitative CEA mRNA detection in peritoneal washes to predict peritoneal relapse based on the predetermined cutoff value. The eligibility criteria consisted of (1) histologically proven gastric cancer, (2) cancer resected without residual disease, (3) absent peritoneal metastases at laparotomy, (4) absent liver metastases at laparotomy, (5) no other active malignancy and (6) provision of written informed consent. Eighty-six patients who underwent potentially curative resection were enrolled between July 2000 and December 2002. This study was approved by the institutional review board of our hospital. Furthermore, the study was also approved by the Ministry of Health, Labor and Warfare of Japan as a highly advanced medical technology specialising in the genetic diagnosis of solid tumours.

### Peritoneal wash specimens

Aliquots of 100–200 ml of saline were introduced into the Douglas cavity and left subphrenic space at the beginning of each operation and aspirated soon after gentle stirring. One-half of each wash was sent to routine cytopathology with conventional Papanicolaou staining and the other half of the wash was used to measure CEA mRNA levels. Intact cells collected from the washes by centrifugation at 1800 r.p.m. for 5 min were rinsed with phosphate-buffered saline (PBS), dissolved in ISOGEN-LS RNA extraction buffer (Nippon Gene, Tokyo, Japan) and stored at −80°C.

### Real-time quantitative reverse transcriptase–polymerase chain reaction

Frozen samples in ISOGEN-LS were thawed and total RNA was extracted using guanidinium isothiocyanate–phenol–chloroform. cDNA was synthesised from total RNA using SuperScript II RNase H^−^ reverse transcriptase (Invitrogen, Carlsbad, CA, USA) according to the manufacturer's instruction. The resultant first-strand cDNA was stored at −80°C until analysis. Single-step real-time RT–PCR for CEA mRNA was performed using CEA-specific oligonucleotide primers and two fluorescent hybridisation probes on the LightCycler instrument (Roche Diagnostics, Mannheim, Germany) as described previously (Nakanishi *et al*, 2000; Kodera *et al*, 2002). To quantify and prove the integrity of the isolated RNA, glyceraldehyde-3-phosphate dehydrogenase (GAPDH) was also analysed by real-time RT–PCR using the appropriate primers and hybridisation probes. All primers and probes were synthesised and purified by reverse-phase HPLC at Nihon Gene Research Laboratories (Sendai, Japan).

Six external CEA mRNA standards were prepared by 10-fold serial dilution (1–10^5^ cells) of cDNA equivalent to 1 × 10^6^ COLM-2 (a colon cancer cell line that expresses large amounts of CEA) cells spiked into 1 × 10^7^ peripheral blood leucocytes. Each run consisted of six external standards, a negative control without a template and patient samples with unknown mRNA concentrations. The amount of mRNA in each sample was then automatically measured by reference to the standard curve constructed each time on the LightCycler software. The higher CEA mRNA value of two washes (Douglas cavity and subphrenic space) from each patient was selected. If at least one CEA mRNA value from the two washes was above the cutoff value, the patient was considered positive for CEA mRNA.

### Data management

The CEA mRNA levels in peritoneal washes were measured by investigators who were blinded to the clinical information of the patients. The results were reported as positive or negative judgments together with relative CEA mRNA values within 2 weeks of sampling. For research purposes, all of the CEA mRNA and clinical data including follow-up information indexed by a unique subject number were given to the chief investigator for further analysis. Tumours were staged according to the TNM classification (5th edition, 1997) and the categories were determined from pathological findings based on surgically resected specimens.

### Postoperative surveillance of patients

Postoperative surveillance proceeded according to the prospective follow-up protocol for individual patients until September 2004 with a median follow-up period of 30 months (range, 21–50 months). The protocol consisted of interim history, physical examination, haematology and blood chemistry panels including tests for CEA and CA19-9, performed every 3 months for the first postoperative year and every 6 months thereafter. The patients were examined by either abdominal ultrasonography or computed tomography every 6 months. Peritoneal recurrence evident according to clinical symptoms, digital examination and physical as well as radiological findings of bowel obstruction and ascites was confirmed by paracentesis, laparotomy and autopsy performed at the discretion of the surgeon.

### Statistical analysis

Receiver-operating characteristic (ROC) curve determined the cutoff value of CEA mRNA as reported (Zweig and Campbell, 1993; Kodera *et al*, 2002). Briefly, ROC curve was constructed by plotting all possible sensitivity/1−specificity pairs resulting from continuously elevating the cutoff values over the range from 0 to 40 000. Sensitivity in this context was defined as a positive rate of CEA mRNA determined during postoperative surveillance, in peritoneal washes from patients with relapses in the form of peritoneal carcinomatosis. Specificity was defined as the negative rate of CEA mRNA among patients without signs of peritoneal recurrence during postoperative surveillance. The CEA mRNA values among each pT category were compared using the Kruskal–Wallis test. Survival was analysed by Kaplan–Meier curves with death and a clinical diagnosis of peritoneal recurrence as end points. Cancer deaths resulting from other types of metastasis in the absence of clinical signs of peritoneal recurrence were treated as censored. Multivariate analysis using the Cox regression hazards model identified independent prognostic factors. Tumour size, histological type, serosal invasion and lymph node metastasis were selected as covariates, along with CEA mRNA status.

## RESULTS

### Clinicopathological characteristics of patients in training and validation sets

Table 1 summarises the characteristics of the patients with gastric cancer who were enrolled in the training (retrospective study) and validation (prospective study) sets. All of the patients underwent curative surgery with R0 resection, but the variables considerably differed between the training and validation sets. The validation set contained significantly more patients with advanced disease than the training set in terms of T (*P*=0.0003) and N (*P*<0.0001) categories. The incidence of patients with advanced gastric cancer (T2–T4) in the training and validation sets was 53.8 and 77.9%, respectively. The incidence of node-positive patients in the training and validation sets was 46.2 and 73.3%, respectively. In contrast, the incidence of patients with positive peritoneal wash cytology was much lower in the validation set (1.2%) than in the training set (9.1%).

### The CEA mRNA level in the peritoneal washes

Real-time RT–PCR using the LightCycler allowed quantitative and sensitive detection of CEA mRNA from patient samples ranging from 1 to 1 × 10^5^ colon carcinoma cells expressing CEA (COLM-2). Carcinoembryonic antigen mRNA was undetectable in peritoneal washes from eight patients with benign disease and in peripheral blood from 15 healthy volunteers as reported (Nakanishi *et al*, 2000). We also confirmed the integrity of extracted RNA with the quantitation of internal control GAPDH mRNA and omitted two of the 256 samples in which GAPDH mRNA was undetectable in the training set (data not shown).

The cutoff value of the CEA mRNA was determined by reference to the ROC curve obtained from 197 patients in the training set. We selected 0.1 as the CEA mRNA cutoff value to allow maximal sensitivity (89.7%) with a minimal false-positive fraction (17.3%).

**Figure 1:**
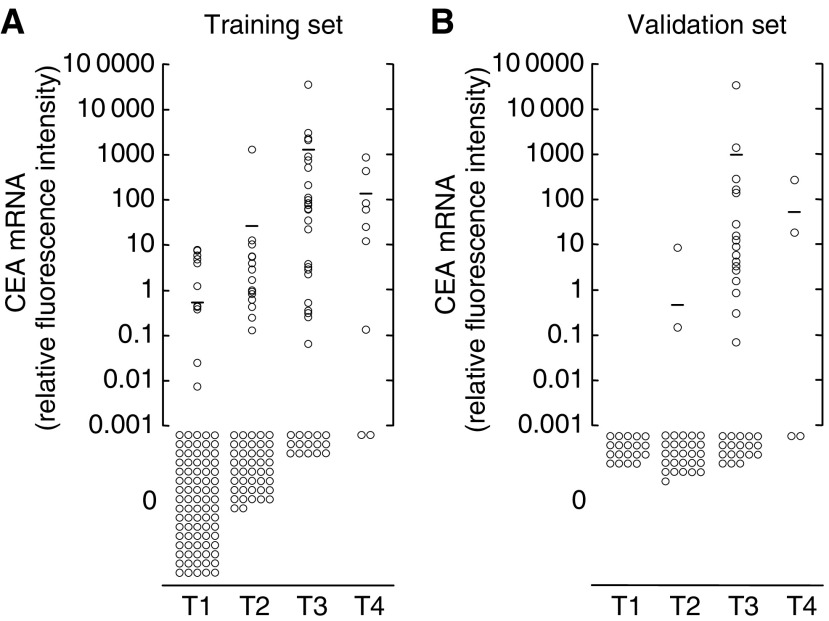
Relative CEA mRNA values of peritoneal washes from gastric cancer patients measured by real-time RT–PCR according to the depth of cancer invasion (pT category) in the training set (**A**) and the validation set (**B**). The CEA mRNA values correlate statistically with depth of invasion in both sets (*P*<0.0001). Bars indicate mean CEA mRNA values.

Figure 1 shows the CEA mRNA expression levels of patients with gastric cancer in the training and validation sets according to the depth of tumour invasion (T category). The CEA mRNA values correlated with the depth of tumour invasion in both training and validation sets (*P*<0.0001) (Figure 1). In the training set, average CEA mRNA values in the peritoneal washes were 0.35, 23, 1119 and 164 for pT1, pT2, pT3 and pT4 tumours, respectively. The positive rates for CEA mRNA were 9.9% (nine out of 91), 26.3% (15 out of 57), 60.0% (24 out of 40) and 77.8% (seven out of nine) in T1, T2, T3 and T4 patients, respectively. In the validation set, average CEA mRNA values were 0, 0.31, 1035 and 71 in patients with T1, T2, T3 and T4 tumours, respectively. The positive rates for CEA mRNA were 0% (0 out of 19), 7.1% (two out of 28), 45.7% (16 out of 35) and 50.0% (two out of four) in T1, T2, T3 and T4 patients, respectively.

### Relationship between survival and CEA mRNA expression

**Figure 2:**
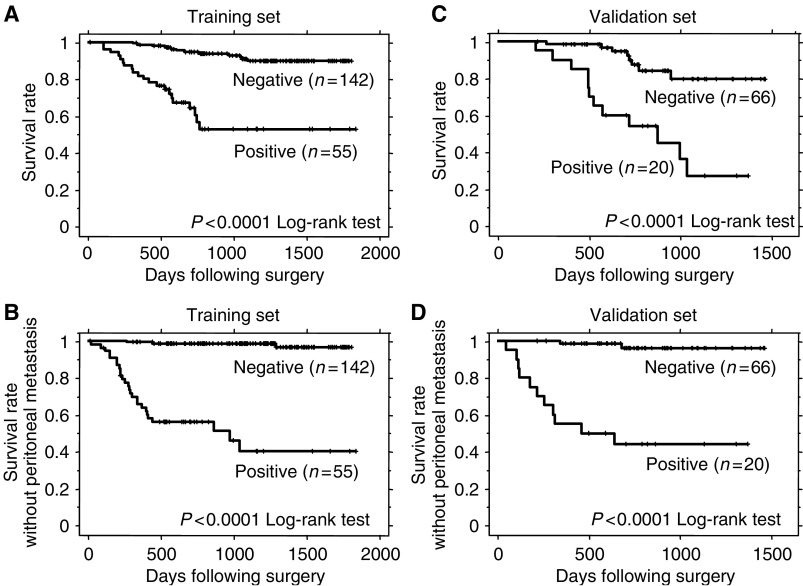
Survival curves of patients with positive and negative CEA mRNA in peritoneal washes in the training set (**A**, **B**) and validation set (**C**, **D**). Panels (**A**, **C**) and (**B**, **D**) show the overall survival and peritoneal recurrence-free survival as end points, respectively. Patients positive and negative for CEA mRNA in peritoneal washes significantly differed (*P*<0.0001, log-rank test) in both training and validation sets.

In the training set, overall survival as well as peritoneal recurrence-free survival among the 197 patients was significantly worse for those with positive CEA mRNA (*P*<0.0001) (Figure 2A and B). A positive rate of CEA mRNA in peritoneal washes from patients with relapses in the form of peritoneal carcinomatosis during postoperative surveillance was 89.7%. Using the cutoff value determined by the training set, we estimated the survival of 86 patients in the validation set. During follow-up until September 2004, 20 patients died from peritoneal (*n*=13), lymph node (*n*=7), bone (*n*=2) and liver (*n*=2) metastases. Twenty of 86 patients (23.3%) were positive for CEA mRNA expression and 12 of these 20 (60.0%) patients died from cancer, but only eight of the remaining 66 (12.1%) who were CEA mRNA-negative died. Overall survival in the 86 patients was significantly worse for those with positive CEA mRNA (*P*<0.0001) (Figure 2C). Among the 86 patients, 13 (15.1%) developed peritoneal metastases. Eleven of the 20 CEA mRNA-positive patients (55.0%) developed peritoneal recurrence, but only two of the remaining 66 patients (3.0%) who were negative for CEA mRNA developed peritoneal disease. Peritoneal recurrence-free survival was also significantly worse in patients with positive CEA mRNA (*P*<0.0001) (Figure 2D). A positive rate of CEA mRNA in peritoneal washes from patients with relapses in the form of peritoneal carcinomatosis during postoperative surveillance was 84.6%.

### Multivariate analysis of prognostic factors in the prospective study

A Cox regression analysis with overall survival as the end point identified independent prognostic factors among covariates including CEA mRNA status, tumour size, histological type, serosal invasion and lymph node metastasis. Carcinoembryonic antigen mRNA along with serosal invasion was an independent prognostic factor for the 86 patients with gastric carcinoma who underwent curative resection (*P*=0.0130) (Table 2). Multivariate analysis with peritoneal recurrence-free survival as the end point revealed only CEA mRNA as an independent prognostic factor (*P*=0.0006) (Table 3).

## DISCUSSION

The present prospective study confirmed that the prognosis was significantly worse for patients with positive CEA mRNA than negative. Furthermore, multivariate analysis identified positive CEA mRNA as an independent prognostic factor. Since we described the quantitative detection of free tumour cells in peritoneal washes (Nakanishi *et al*, 2000) and its prognostic significance for gastric cancer patients (Kodera *et al*, 2002), many investigators have reported the usefulness of quantitative CEA mRNA detection in retrospective risk assessments of peritoneal recurrence (Nakanishi *et al*, 2004). To our knowledge, the present study is the first prospective validation of quantitative CEA mRNA detection in peritoneal washes as a reliable prognostic indicator of peritoneal recurrence in the clinical setting for gastric cancer patients.

Quantitative detection of CEA mRNA has been a matter of some controversy, such as determination of the cutoff value and whether or not to correct CEA mRNA values using an internal standard. In our preliminary study as well as those of others (Oyama *et al*, 2004), the cutoff value was determined based on the mean+2 s.d. (standard deviation) of control samples derived from patients who underwent surgery to treat benign diseases. Since CEA mRNA was undetectable in the control samples in our system, the calculated cutoff value would be zero, resulting in a high incidence of false-positive results and low specificity. To avoid an arbitrary selection, we performed ROC curve analyses to determine the optimal cutoff value of CEA mRNA that could adequately evaluate peritoneal recurrence risk. We selected a cutoff value of 0.1 to give maximal sensitivity with a minimal false-positive fraction. Resultant sensitivity and specificity were 89.7 and 82.7% in the training set and 84.6 and 87.7% in the validation set, respectively. The cutoff value of 0.1 was determined without reference to the data from the validation set; nevertheless, the sensitivity and specificity were almost equivalent between the two data sets, indicating the reproducibility of the diagnostic ability of real-time CEA RT–PCR. Whether to use a simple CEA mRNA value or the value corrected with the internal control as a predictive indicator of peritoneal recurrence is another controversial issue. Oyama *et al* (2004) recommended using the CEA/GAPDH ratio from the viewpoint of reliability. However, our previous comparison of the prognostic values of CEA mRNA and the CEA/GAPDH ratio demonstrated that the area under the ROC curves for CEA mRNA with or without correction by GAPDH mRNA were essentially the same, indicating that correction with reference to GAPDH mRNA may not be strictly necessary (Kodera *et al*, 2002). We believe that the total number of cancer cells represented by CEA mRNA rather than the ratio of cancer/noncancer cells (CEA/GAPDH ratio) is a more important hallmark for predicting peritoneal recurrence. We therefore used simple, uncorrected CEA mRNA values for subsequent analysis in the present study.

The validation set was characterised in the present study by a greater number of advanced gastric cancer patients in terms of T and N categories who were peritoneal wash cytology-negative, reflecting the patient population that would benefit most from the assessment of peritoneal recurrence risk by quantitative CEA mRNA detection. Even in such a validation set, CEA mRNA proved to be a significant independent prognostic factor by multivariate analysis, with both overall survival and peritoneal recurrence-free survival as the end points. In contrast to CEA mRNA in the peritoneal washes, traditional markers such as depth of invasion and lymph node status were, unexpectedly, not always prognostically significant in the validation set. As for depth of tumour invasion, multivariate analysis identified serosal invasion as an independent prognostic factor when overall survival, but not peritoneal recurrence-free survival, was adopted as the end point. The lack of prognostic significance of serosal invasion in peritoneal recurrence-free survival is probably because the depth of tumour invasion correlates with not only peritoneal recurrence, but also lymph node and/or haematogenous recurrence. These findings suggest that CEA mRNA in the peritoneal washes is a genuine prognostic factor for peritoneal recurrence, being more reliable than serosal invasion (depth of invasion). On the other hand, lymph node metastasis, which is generally known to be one of the most important prognostic factors, was surely a significant prognostic indicator for overall survival with both univariate and multivariate analysis in the training set, whereas surprisingly, lymph node status in the validation set was not a significant prognostic factor even in the univariate analysis. We speculate that this discrepancy between the training set and validation set is at least in part due to the recent advances in chemotherapy for gastric cancer. New-generation agents such as irinotecan, docetaxel, paclitaxel and S-1 (oral DPD inhibitory fluoropyrimidine) have been developed and used for gastric cancer patients with promising antitumour effects (Futatsuki *et al*, 1994; Sakata *et al*, 1998; Koizumi *et al*, 2000; Yamada *et al*, 2001; Bang *et al*, 2002). In fact, S-1 achieved the highest response rate (48%) among these agents, especially for the distant lymph nodes (Sakata *et al*, 1998). Therefore, S-1 was used in more than 80% of patients with recurrence in our validation set, and 2-year survival rate for node-positive patients was improved from 69.7% in the training set to 77.5% in the validation set, suggesting that lymph node metastasis is more controllable than peritoneal metastasis by chemotherapy. In addition, the shorter follow-up period of the validation set than the training set might adversely affect estimation of the prognostic ability of lymph node status.

It is somewhat puzzling that the positive rate of CEA mRNA in the validation set (23.3%) was lower than that of the training set (27.9%), although the validation set presented a higher incidence of patients with ‘classical’ adverse prognostic factors such as serosal invasion and/or lymph node metastases. Similarly, the incidence of cytology-positive patients in the validation set was much lower in the validation set (1.2%) than the training set (8.6%). These discrepancies are considered to be mainly attributable to two reasons. The first reason is our recent change in therapeutic policy from resection to nonresection approach for type IV (scirrhous type) gastric cancer with positive cytology findings or laparoscopic evidence of peritoneal dissemination (Kodera *et al*, 2001). In the same period as the validation set, gastrectomy could be avoided in five scirrhous carcinoma patients with positive cytology findings and another five patients with peritoneal dissemination diagnosed for the first time by laparoscopy. These patients were not eligible for the present study and therefore were excluded from the validation set, resulting in the lower rate of CEA mRNA-positive and cytology-positive patients. The second reason for the low incidence of positive CEA mRNA in the validation set is our technical refinement of CEA mRNA quantitation. The positive rate for CEA mRNA in T1-stage gastric cancer patients is much lower in the validation set (0%) than in the training set (9.9%), indicating a decrease in the false-positive results in the validation set. In the present analysis, we did not add peritoneal wash cytology as a covariate for multivariate analysis because of the low incidence of cytology-positive patients (only one patient) in the validation set. However, we examined and confirmed that CEA mRNA was an independent prognostic factor even in an analysis model including peritoneal wash cytology as a covariate (data not shown).

Micrometastasis has recently been classified into ‘isolated tumour cells (ITC)’, which are single tumour cells or a small cell cluster that is no larger than 0.2 mm at the greatest diameter, and ‘micrometastases’, which are larger than 0.2 mm according to UICC (Sobin, 2003). The two classes should be separated because ITC do not typically show morphological evidence of metastatic activity such as penetration of a vascular or lymph sinus wall, tumour cell growth and stromal reaction (Weaver, 2003; Cserni *et al*, 2005). In fact, several investigators reported that ITC do not become metastatic and will probably die or be eliminated by immune surveillance (Holmgren *et al*, 1995). Quantitative CEA RT–PCR can detect such ITC and, therefore, we assume that the specificity has an upper limitation of possibly 80–90% at maximal incidence. The remaining 10–20% of the CEA mRNA-positive patients without peritoneal recurrence might include those with ITC and false positives.

Micrometastasis is clinically important not only as a prognostic indicator as described above, but also as a potential therapeutic target. A unique feature of micrometastases that distinguishes them from macroscopic metastases is their high sensitivity to anticancer drugs. Several experimental studies have demonstrated a preferential therapeutic efficacy for micrometastases in the lung (Kurebayashi *et al*, 1997) and peritoneum (Chaudhuri *et al*, 2001) compared with macroscopic metastasis. Mice bearing gastric cancer micrometastases in the peritoneal cavity survived longer than those with macroscopic metastases after chemotherapy, and some of them achieved pathological complete regression or were cured (Nakanishi *et al*, 2003). Based on this chemosensitivity, we proposed a new therapeutic strategy for protecting gastric cancer patients from peritoneal recurrence after surgery. The strategy consists of molecular diagnostic detection and subsequent adjuvant chemotherapy targeted towards micrometastases. The present study proved that quantitative CEA RT–PCR is a feasible molecular diagnostic tool that can help to realise such a therapeutic strategy.

In conclusion, we confirmed that quantitative real-time RT–PCR is a powerful means of identifying subgroups of patients at high risk for peritoneal relapse. The selection of high-risk patients seems to be essential for treating gastric cancer patients with individualised therapy. Exploratory phase II clinical trials of oral 5-FU derivatives for real-time RT–PCR-positive gastric cancer patients are now ongoing in our institute.

## Figures and Tables

**Table 1:** Patients characteristics of training set and validation set

**Variate**	**Training set**	**Validation set**	***P-*value**
*Tumour size*			
⩽5 cm	107 (54.3%)	39 (45.3%)	0.1961
>5 cm	90 (45.7%)	47 (54.7%)	
			
*Histological type*			
Differentiated	66 (33.5%)	28 (32.6%)	0.8767
Undifferentiated	131 (66.5%)	58 (67.4%)	
			
*T stage*			
t1	91 (46.2%)	19 (22.1%)	0.0003
t2	57 (28.9%)	28 (32.6%)	
t3	40 (20.3%)	35 (40.7%)	
t4	9 (4.6%)	4 (4.7%)	
			
*Lymph node metastasis*			
Negative	106 (53.8%)	23 (26.7%)	<0.0001
Positive	91 (46.2%)	63 (73.3%)	
			
*Peritoneal wash cytology*			
Class I	100 (50.8%)	78 (90.7%)	<0.0001
Class II	74 (37.6%)	3 (3.5%)	
Class III	5 (2.5%)	4 (4.7%)	
Class IV	1 (0.5%)	0 (0.0%)	
Class V	17 (8.6%)	1 (1.2%)	
			
*CEA mRNA status in peritoneal wash*			
Negative	142 (72.1%)	66 (76.7%)	0.4657
Positive	55 (27.9%)	20 (23.3%)	
			
*Operative type*			
Distal gastrectomy	133 (67.5%)	45 (52.3%)	0.0635
Proximal gastrectomy	12 (6.1%)	6 (6.9%)	
Total gastrectomy	51 (25.9%)	33 (38.4%)	
Pancreaticoduodenectomy	1 (0.5%)	2 (2.3%)	
			
*Adjuvant chemotherapy*			
Yes	41 (20.8%)	11 (12.8%)	0.1334
No	156 (79.2%)	75 (87.2%)	
			

CEA=carcinoembryonic antigen.

**Table 2:** Multivariate analysis of prognostic factors in the validation set with overall survival as an end point

**Variate**	**Hazard ratio**	**95% confidence interval**	***P*-value**
*Tumour size*			
⩽5 cm	1		
>5 cm	0.54	0.19–1.51	0.2400
			
*Histological type*			
Differentiated	1		
Undifferentiated	1.97	0.78–4.97	0.1500
			
*Serosal invasion*			
Negative	1		
Positive	3.38	1.21–9.50	0.0210
			
*Lymph node metastasis*			
Negative	1		
Positive	0.78	0.37–1.65	0.5200
			
*CEA mRNA status*			
Negative	1		
Positive	1.79	1.13–2.85	0.0130

CEA=carcinoembryonic antigen.

**Table 3:** Multivariate analysis of prognostic factors in the validation set with peritoneal recurrence-free survival as an end point

**Variate**	**Hazard ratio**	**95% confidence interval**	***P*-value**
*Tumour size*			
⩽5 cm	1		
>5 cm	0.32	0.06–1.68	0.1800
			
*Histological type*			
Differentiated	1		
Undifferentiated	2.64	0.55–12.70	0.2300
			
*Serosal invasion*			
Negative	1		
Positive	155.09	2.02E−10–1.19E+14	0.7200
			
*Lymph node metastasis*			
Negative	1		
Positive	0.65	0.21–1.97	0.4400
			
*CEA mRNA status*			
Negative	1		
Positive	3.99	1.80–8.84	0.0006

CEA=carcinoembryonic antigen.

## References

[bib1] Abe S, Yoshimura H, Tabara H, Tachibana M, Monden N, Nakamura T, Nagaoka S (1995) Curative resection of gastric cancer: limitation of peritoneal lavage cytology in predicting the outcome. J Surg Oncol 59: 226–229 7630168 10.1002/jso.2930590405

[bib2] Bando E, Yonemura Y, Takeshita Y, Taniguchi K, Yasui T, Yoshimitsu Y, Fushida S, Fujimura T, Nishimura G, Miwa K (1999) Intraoperative lavage for cytological examination in 1,297 patients with gastric carcinoma. Am J Surg 178: 256–262 10527450 10.1016/s0002-9610(99)00162-2

[bib3] Bang YJ, Kang WK, Kang YK, Kim HC, Jacques C, Zuber E, Daglish B, Boudraa Y, Kim WS, Heo DS, Kim NK (2002) Docetaxel 75 mg/m(2) is active and well tolerated in patients with metastatic or recurrent gastric cancer: a phase II trial. Jpn J Clin Oncol 32: 248–254 12324575 10.1093/jjco/hyf057

[bib4] Benevolo M, Mottolese M, Cosimelli M, Tedesco M, Giannarelli D, Vasselli S, Carlini M, Garofalo A, Natali PG (1998) Diagnostic and prognostic value of peritoneal immunocytology in gastric cancer. J Clin Oncol 16: 3406–3411 9779720 10.1200/JCO.1998.16.10.3406

[bib5] Boku T, Nakane Y, Minoura T, Takada H, Yamamura M, Hioki K, Yamamoto M (1990) Prognostic significance of serosal invasion and free intraperitoneal cancer cells in gastric cancer. Br J Surg 77: 436–439 2340396 10.1002/bjs.1800770425

[bib6] Bonenkamp JJ, Songun I, Hermans J, van de Velde CJ (1996) Prognostic value of positive cytology findings from abdominal washings in patients with gastric cancer. Br J Surg 83: 672–674 8689216 10.1002/bjs.1800830526

[bib7] Chaudhuri TR, Mountz JM, Rogers BE, Partridge EE, Zinn KR (2001) Light-based imaging of green fluorescent protein-positive ovarian cancer xenografts during therapy. Gynecol Oncol 82: 581–589 11520161 10.1006/gyno.2001.6297

[bib8] Cserni G, Bianchi S, Boecker W, Decker T, Lacerda M, Rank F, Wells CA (2005) Improving the reproducibility of diagnosing micrometastases and isolated tumor cells. Cancer 103: 358–367 15593354 10.1002/cncr.20760

[bib9] Fujii S, Kitayama J, Kaisaki S, Sasaki S, Seto Y, Tominaga O, Tsuno N, Umetani N, Yokota H, Kitamura K, Tsuruo T, Nagawa H (2002) Carcinoembryonic antigen mRNA in abdominal cavity as a useful predictor of peritoneal recurrence of gastric cancer with serosal exposure. J Exp Clin Cancer Res 21: 547–553 12636101

[bib10] Futatsuki K, Wakui A, Nakao I, Sakata Y, Kambe M, Shimada Y, Yoshino M, Taguchi T, Ogawa N (1994) Late phase II study of irinotecan hydrochloride (CPT-11) in advanced gastric cancer. Gan To Kagaku Ryoho 21: 1033–1038 8210254

[bib11] Gillanders WE, Mikhitarian K, Hebert R, Mauldin PD, Palesch Y, Walters C, Urist MM, Mann GB, Doherty G, Herrmann VM, Hill AD, Eremin O, El-Sheemy M, Orr RK, Valle AA, Henderson MA, Dewitty RL, Sugg SL, Frykberg E, Yeh K, Bell RM, Metcalf JS, Elliott BM, Brothers T, Robison J, Mitas M, Cole DJ (2004) Molecular detection of micrometastatic breast cancer in histopathology-negative axillary lymph nodes correlates with traditional predictors of prognosis: an interim analysis of a prospective multi-institutional cohort study. Ann Surg 239: 828–837 15166962 10.1097/01.sla.0000128687.59439.d6 PMC1356291

[bib12] Hayes N, Wayman J, Wadehra V, Scott DJ, Raimes SA, Griffin SM (1999) Peritoneal cytology in the surgical evaluation of gastric carcinoma. Br J Cancer 79: 520–524 10027323 10.1038/sj.bjc.6690081 PMC2362407

[bib13] Holmgren L, O’Reilly MS, Folkman J (1995) Dormancy of micrometastases: balanced proliferation and apoptosis in the presence of angiogenesis suppression. Nat Med 1: 149–153 7585012 10.1038/nm0295-149

[bib14] Jotsuka T, Okumura Y, Nakano S, Nitta H, Sato T, Miyachi M, Suzumura K, Yamashita J (2004) Persistent evidence of circulating tumor cells detected by means of RT–PCR for CEA mRNA predicts early relapse: a prospective study in node-negative breast cancer. Surgery 135: 419–426 15041966 10.1016/j.surg.2003.08.014

[bib15] Juhl H, Stritzel M, Wroblewski A, Henne-Bruns D, Kremer B, Schmiegel W, Neumaier M, Wagener C, Schreiber HW, Kalthoff H (1994) Immunocytological detection of micrometastatic cells: comparative evaluation of findings in the peritoneal cavity and the bone marrow of gastric, colorectal and pancreatic cancer patients. Int J Cancer 57: 330–335 8168992 10.1002/ijc.2910570307

[bib16] Kodera Y, Nakanishi H, Ito S, Yamamura Y, Kanemitsu Y, Shimizu Y, Hirai T, Yasui K, Kato T, Tatematsu M (2002) Quantitative detection of disseminated free cancer cells in peritoneal washes with real-time reverse transcriptase–polymerase chain reaction: a sensitive predictor of outcome for patients with gastric carcinoma. Ann Surg 235: 499–506 11923605 10.1097/00000658-200204000-00007 PMC1422464

[bib17] Kodera Y, Nakanishi H, Yamamura Y, Shimizu Y, Torii A, Hirai T, Yasui K, Morimoto T, Kato T, Kito T, Tatematsu M (1998) Prognostic value and clinical implications of disseminated cancer cells in the peritoneal cavity detected by reverse transcriptase–polymerase chain reaction and cytology. Int J Cancer 79: 429–433 9699538 10.1002/(sici)1097-0215(19980821)79:4<429::aid-ijc20>3.0.co;2-z

[bib18] Kodera Y, Yamamura Y, Ito S, Kanemitsu Y, Shimizu Y, Hirai T, Yasui K, Kato T (2001) Is Borrmann type IV gastric carcinoma a surgical disease? An old problem revisited with reference to the result of peritoneal washing cytology. J Surg Oncol 78: 175–181 11745801 10.1002/jso.1144

[bib19] Kodera Y, Yamamura Y, Shimizu Y, Torii A, Hirai T, Yasui K, Morimoto T, Kato T (1999) Peritoneal washing cytology: prognostic value of positive findings in patients with gastric carcinoma undergoing a potentially curative resection. J Surg Oncol 72: 60–64 10518099 10.1002/(sici)1096-9098(199910)72:2<60::aid-jso3>3.0.co;2-1

[bib20] Koizumi W, Kurihara M, Nakano S, Hasegawa K (2000) Phase II study of S-1, a novel oral derivative of 5-fluorouracil, in advanced gastric cancer. For the S-1 Cooperative Gastric Cancer Study Group. Oncology 58: 191–197 10765119 10.1159/000012099

[bib21] Kurebayashi J, Nukatsuka M, Fujioka A, Saito H, Takeda S, Unemi N, Fukumori H, Kurosumi M, Sonoo H, Dickson RB (1997) Postsurgical oral administration of uracil and tegafur inhibits progression of micrometastasis of human breast cancer cells in nude mice. Clin Cancer Res 3: 653–659 9815733

[bib22] Marutsuka T, Shimada S, Shiomori K, Hayashi N, Yagi Y, Yamane T, Ogawa M (2003) Mechanisms of peritoneal metastasis after operation for non-serosa-invasive gastric carcinoma: an ultrarapid detection system for intraperitoneal free cancer cells and a prophylactic strategy for peritoneal metastasis. Clin Cancer Res 9: 678–685 12576435

[bib23] Nakanishi H, Kodera Y, Tatematsu M (2004) Molecular method to quantitatively detect micrometastases and its clinical significance in gastrointestinal malignancies. Adv Clin Chem 38: 87–110 15521189 10.1016/s0065-2423(04)38003-0

[bib24] Nakanishi H, Kodera Y, Torii A, Hirai T, Yamamura Y, Kato T, Kito T, Tatematsu M (1997) Detection of carcinoembryonic antigen-expressing free tumor cells in peritoneal washes from patients with gastric carcinoma by polymerase chain reaction. Jpn J Cancer Res 88: 687–692 9310142 10.1111/j.1349-7006.1997.tb00437.x PMC5921487

[bib25] Nakanishi H, Kodera Y, Yamamura Y, Ito S, Kato T, Ezaki T, Tatematsu M (2000) Rapid quantitative detection of carcinoembryonic antigen-expressing free tumor cells in the peritoneal cavity of gastric-cancer patients with real-time RT–PCR on the lightcycler. Int J Cancer 89: 411–417 11008202 10.1002/1097-0215(20000920)89:5<411::aid-ijc3>3.0.co;2-5

[bib26] Nakanishi H, Mochizuki Y, Kodera Y, Ito S, Yamamura Y, Ito K, Akiyama S, Nakao A, Tatematsu M (2003) Chemosensitivity of peritoneal micrometastases as evaluated using a green fluorescence protein (GFP)-tagged human gastric cancer cell line. Cancer Sci 94: 112–118 12708484 10.1111/j.1349-7006.2003.tb01361.x PMC11160247

[bib27] Oyama K, Terashima M, Takagane A, Maesawa C (2004) Prognostic significance of peritoneal minimal residual disease in gastric cancer detected by reverse transcription–polymerase chain reaction. Br J Surg 91: 435–443 15048743 10.1002/bjs.4455

[bib28] Sakata Y, Ohtsu A, Horikoshi N, Sugimachi K, Mitachi Y, Taguchi T (1998) Late phase II study of novel oral fluoropyrimidine anticancer drug S-1 (1 Mtegafur–0.4 Mgimestat–1 Motastat potassium) in advanced gastric cancer patients. Eur J Cancer 34: 1715–1720 9893658 10.1016/s0959-8049(98)00211-1

[bib29] Sobin LH (2003) TNM, sixth edition: new developments in general concepts and rules. Semin Surg Oncol 21: 19–22 12923912 10.1002/ssu.10017

[bib30] Tokuda K, Natsugoe S, Nakajo A, Miyazono F, Ishigami S, Hokita S, Takao S, Eizuru Y, Aikou T (2003) Clinical significance of CEA-mRNA expression in peritoneal lavage fluid from patients with gastric cancer. Int J Mol Med 11: 79–84 12469223

[bib31] Ueno H, Yoshida K, Hirai T, Kono F, Kambe M, Toge T (2003) Quantitative detection of carcinoembryonic antigen messenger RNA in the peritoneal cavity of gastric cancer patients by real-time quantitative reverse transcription polymerase chain reaction. Anticancer Res 23: 1701–1708 12820444

[bib32] Weaver DL (2003) Sentinel lymph nodes and breast carcinoma: which micrometastases are clinically significant? Am J Surg Pathol 27: 842–845 12766591 10.1097/00000478-200306000-00018

[bib33] Yamada Y, Shirao K, Ohtsu A, Boku N, Hyodo I, Saitoh H, Miyata Y, Taguchi T (2001) Phase II trial of paclitaxel by three-hour infusion for advanced gastric cancer with short premedication for prophylaxis against paclitaxel-associated hypersensitivity reactions. Ann Oncol 12: 1133–1137 11583196 10.1023/a:1011680507956

[bib34] Yonemura Y, Endou Y, Fujimura T, Fushida S, Bandou E, Kinoshita K, Sugiyama K, Sawa T, Kim BS, Sasaki T (2001) Diagnostic value of preoperative RT–PCR-based screening method to detect carcinoembryonic antigen-expressing free cancer cells in the peritoneal cavity from patients with gastric cancer. Aust NZ J Surg 71: 521–528 10.1046/j.1440-1622.2001.02187.x 11527261

[bib35] Zweig MH, Campbell G (1993) Receiver-operating characteristic (ROC) plots: a fundamental evaluation tool in clinical medicine. Clin Chem 39: 561–577 8472349

